# The Effect of DNA Methylation in the Development and Progression of Chronic Kidney Disease in the General Population: An Epigenome-Wide Association Study Using the Korean Genome and Epidemiology Study Database

**DOI:** 10.3390/genes14071489

**Published:** 2023-07-21

**Authors:** Ji-Eun Kim, Min-Jee Jo, Eunjung Cho, Shin-Young Ahn, Young-Joo Kwon, Jeong-An Gim, Gang-Jee Ko

**Affiliations:** 1 Department of Internal Medicine, Korea University Guro Hospital, Seoul 08308, Republic of Korea; beeswaxag@naver.com (J.-E.K.); minjeeyoyo@naver.com (M.-J.J.); icdej@naver.com (E.C.); ahnshinyoung712@gmail.com (S.-Y.A.); yjkwon@korea.ac.kr (Y.-J.K.); 2 Medical Science Research Center, Korea University College of Medicine, Korea University Guro Hospital, Seoul 08308, Republic of Korea

**Keywords:** chronic kidney disease, DNA methylation, NPHS2, CHCHD4

## Abstract

Background: Although knowledge of the genetic factors influencing kidney disease is increasing, epigenetic profiles, which are associated with chronic kidney disease (CKD), have not been fully elucidated. We sought to identify the DNA methylation status of CpG sites associated with reduced kidney function and examine whether the identified CpG sites are associated with CKD development. Method: We analyzed DNA methylation patterns of 440 participants in the Korean Genome and Epidemiology Study (KoGES) with estimated glomerular filtration rates (eGFRs) ≥ 60 mL/min/1.73 m^2^ at baseline. CKD development was defined as a decrease in the eGFR of <60 at any time during an 8-year follow-up period (“CKD prediction” analysis). In addition, among the 440 participants, 49 participants who underwent a second methylation profiling were assessed for an association between a decline in kidney function and changes in the degree of methylation of CpG sites during the 8 years (“kidney function slope” analysis). Results: In the CKD prediction analysis, methylation profiles of a total of 403,129 CpG sites were evaluated at baseline in 440 participants, and increased and decreased methylation of 268 and 189 CpG sites, respectively, were significantly correlated with the development of CKD in multivariable logistic regression. During kidney function slope analysis using follow-up methylation profiles of 49 participants, the percent methylation changes in 913 CpG sites showed a linear relationship with the percent change in eGFR during 8 years. During functional enrichment analyses for significant CpG sites found in the CKD prediction and kidney function slope analyses, we found that those CpG sites represented MAPK, PI3K/Akt, and Rap1 pathways. In addition, three CpG sites from three genes, *NPHS2*, *CHCHD4*, and *AHR*, were found to be significant in the CKD prediction analysis and related to a decline in kidney function. Conclusion: It is suggested that DNA methylation on specific genes is associated with the development of CKD and the deterioration of kidney function.

## 1. Introduction

Chronic kidney disease (CKD) is a global public health problem. CKD affects 10–15% of the population worldwide and is now recognized as the most rapidly increasing contributor to the global burden of disease [1,2]. CKD can be defined as the sustained presence of a reduced glomerular filtration rate [3]. It is widely recognized that CKD plays a significant role in the development of cardiovascular disease, metabolic disorders, hospitalization, and mortality [4,5,6]. However, despite the clinical significance of CKD, our understanding of the biological mechanisms underlying CKD and its progression remains incomplete. Traditional biomarkers of CKD, including creatinine and proteinuria, are reliable and easily measured with non-invasive methods. However, their ability to detect kidney injury in early stages and identify populations at high risk of CKD progression is limited [7]. Identifying CKD at earlier stages and recognizing the risk of progression are the key ways to manage and prevent CKD. A lot of studies revealing novel biomarkers for kidney disease have been conducted [8,9].

CKD is a multifactorial disease associated with various genetic and environmental risk factors [10]. Recent large-scale genome-wide association studies (GWAS) have discovered hundreds of common variants associated with CKD risk and kidney function [11,12]. While these GWAS provide insights into the genetic background associated with the pathophysiology of CKD, they cannot detect longitudinal changes or the environmental effect. Instead, epigenomic and transcriptomic profiling are required to obtain the complete picture of CKD regarding the environmental effect. 

DNA methylation is one of the main epigenetic mechanisms for the regulation of gene expression [13,14]. Most CpG islands are found in promoter regions and closely linked to gene expression. Generally, when CpG islands undergo methylation, they block the access of several transcription factors involved in gene activation, resulting in the suppression of gene expression [15]. 

Previous studies on methylation in association with CKD have been limited by factors such as small sample sizes, cross-sectional designs, or inclusion of individuals with specific comorbidities [16,17,18,19]. In 2011, a large cohort was analyzed in an epigenetics study of CKD [17]. However, the definition of CKD in this study was based solely on estimated glomerular filtration rates (eGFR) <60 mL/min/1.73 m^2^ measured at a single time point, which poses challenges in accurately distinguishing it as “chronic” kidney injury. Additionally, no previous methylation studies focused on examining temporal changes in methylation patterns specifically related to renal function.

This study addresses the limitations of previous research by introducing a novel approach to defining renal dysfunction. We consider both the estimated glomerular filtration rate (eGFR) levels at a single time point and the rate of change in renal function during long-term follow-up. Additionally, we analyze the associated changes in methylation patterns. Our goal is to identify methylation sites that substantially correlate with renal function decline, as well as the development of CKD. Consequently, our study aims to identify specific genes that can be targeted for future epigenetic biomarker analysis, thereby providing valuable insights into the field of kidney research. 

## 2. Materials and Methods

### 2.1. Study Population

The present study was approved by the institutional review board (IRB) of Korea University Guro Hospital (IRB no. 2020-0191-01). All participants were recruited from the Korean Genome Epidemiology Study (KoGES), a longitudinal community-based prospective study initiated in 2001–2002 with follow-up performed every 2 years until the eighth follow-up at 16 years. DNA methylation profiling was performed in the baseline study and reanalyzed at the fifth follow-up point after 8 years. The KoGES cohort consists of Korean middle-aged general population participants aged between 40 and 69 years. The details of the study protocol concerning the enrollment and follow-up of participants were presented previously [20]. 

### 2.2. DNA Methylation Profiling

For DNA methylation profiling, genomic DNA was extracted from the buffy coats of participants isolated within 2 h of blood collection. The sample collection time consisted of two times: at the time of enrollment in the KoGES study (n = 446) and follow-up after 8 years (n = 50). DNA methylation was measured with the Illumina Infinium Human Methylation 450 BeadChip system (Illumina, San Diego, CA, USA), which interrogates >485,000 CpGs covering 99% of RefSeq genes. The DNA methylation assessment procedure has been described previously [21,22]. In brief, genomic DNA (500-ng for each sample) was modified by sodium bisulfite, using an EZ DNA methylation kit (Zymo Research, Orange, CA, USA) according to manufacturer’s recommendations. Additionally, the extraction of the intensity values of each site, along with background correction, was carried out using the GenomeStudio V2011 (Methylation Module, R 2.11) software after passing quality-control steps. Each methylation datapoint was identified by fluorescent signals, and the β-value was calculated to signify percent methylation, from 0 to 100%, by the ratio of signals from methylated and unmethylated alleles. CpGs with more than 1% missing data across all samples were discarded.

### 2.3. Analysis Regarding Incidence and Progression of CKD

The demographic and clinical characteristics of the participants, including age, sex, height, weight, and history of hypertension and diabetes were collected. Laboratory parameters such as serum blood urea nitrogen, creatinine, and albumin were also measured at baseline and follow-up visits. The kidney function of participants was evaluated using eGFR, which was calculated with the Chronic Kidney Disease Epidemiology Collaboration (CKD-EPI) equation [23]. It takes into account variables such as age, gender, race, and serum creatinine levels to predict eGFR. The CKD-EPI equation, expressed as a single equation, is eGFR = 141 × min(Scr/κ, 1)^α^ × max(Scr/κ, 1)^−1.209^ × 0.993^Age^ × 1.018 [if female] × 1.159 [if black], where Scr is serum creatinine, κ is 0.7 for women and 0.9 for men, α is −0.329 for women and −0.411 for men, min indicates the minimum of Scr/κ or 1, and max indicates the maximum of Scr/κ or 1. The development of CKD was defined as a decline of eGFR to <60 mL/min/1.73 m^2^ at any time point in 8 years.

**Figure 1:**
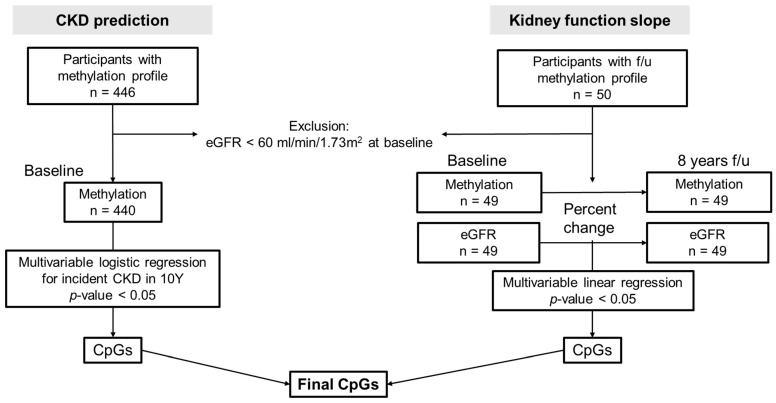
Study design. This study consisted of 2 analysis models; “CKD prediction” was the logistic regression analysis for CKD development in 8 years and “kidney function slope” was the linear regression analysis between changes in methylation scores and changes in eGFR for 8 years. Both analyses were performed with multivariable adjustment, including age, sex, baseline eGFR, hypertension, diabetes, and serum albumin level. The final aim of this study was to find the common CpGs and related genes in both analyses. 10Y, 10 years; f/u, follow-up.

We performed an assessment of the association between methylation profiles and the decline in kidney function using two separated analyses. First, the logistic regression analysis for the risk of CKD development in 8 years was performed with baseline methylation profiles (“CKD prediction” analysis). The logistic regression model estimates the probability of the dependent variable belonging to a particular category or group based on the values of the independent variables. This model can provide insights into the factors associated with the likelihood of CKD occurrence. Then, the analysis was performed with the linear regression analysis between the percent decline in eGFR and the percent change in β-value in the methylation profile between baseline and follow-up methylation profiles (“kidney function slope” analysis). The relationship between the longitudinal changes in eGFR and methylation status was assessed using linear regression. A flow diagram of the study design is represented in Figure 1. To clarify the progression of kidney dysfunction, we excluded participants with a eGFR < 60 mL/min/1.73 m^2^ at baseline. 

### 2.4. Statistical Analysis and Kyoto Encyclopedia of Genes and Genomes (KEGG) Pathway Analysis

In baseline characteristics, categorical variables are presented as numbers and percentages and continuous variables are presented as the mean ± standard deviation (SD) values. To predict the risk of CKD development in 8 years, univariable and multivariable logistic regression analyses were performed. The relationship between changes in eGFR and changes in methylation profiles was also examined by univariable and multivariable linear regression analyses. In the multivariable regression analysis, age, sex, hypertension, diabetes, and eGFR were used as adjustment variables. All significant CpGs found in logistic regression (CKD prediction analysis) and linear regression (kidney function slope analysis) were represented with genomic information by annotating with the “IlluminaHumanMethylation450-kanno.ilmn12.hg19” R package, “getAnnotation” function. 

We performed KEGG pathway analyses of the differentially methylated genes with clusterProfiler version 4.0.5 and the pathview R package. KEGG is an encyclopedia of genes and genomes [24]. Molecular functions are represented by networks of interactions and reactions mainly in the form of KEGG pathways and modules. The clusterProfiler package provides a function, enrichKEGG, to calculate enrichment values for KEGG pathways based on hypergeometric distribution [25]. In dotplots for KEGG pathways, the gene ratio (i.e., ratio of input genes that are annotated in a term) was indicated for top features in the enrichment test. The “circlize” R package (version 0.4.14) was used for the circos plot representing the genetic mapping of significant CpGs found in analyses. 

*p* < 0.001 and false-discovery rate (FDR)-adjusted *p* (q-value) < 0.05 were considered statistically significant in the univariable and multivariable analyses, respectively [26,27,28,29]. All statistical analyses were performed using the R software program (version 4.0.3; R Foundation for Statistical Computing, Vienna, Austria).

## 3. Results

### 3.1. Baseline Characteristics 

Of the 446 participants with baseline methylation profiles, 6 participants with an estimated glomerular filtration rate (eGFR) < 60 mL/min/1.73 m^2^ at baseline were excluded. The baseline characteristics of the remaining 440 participants are shown in Table 1. The mean age of participants was 52.1 ± 8.4 years, and 48.9% were female. The baseline creatinine and eGFR values were 0.85 ± 0.17 mg/dL and 91.8 ± 12.8 mL/min/1.73 m^2^, respectively. 

### 3.2. Baseline Methylation Profiles Associated with CKD Development in 8 Years (CKD Prediction Analysis)

**Figure 2:**
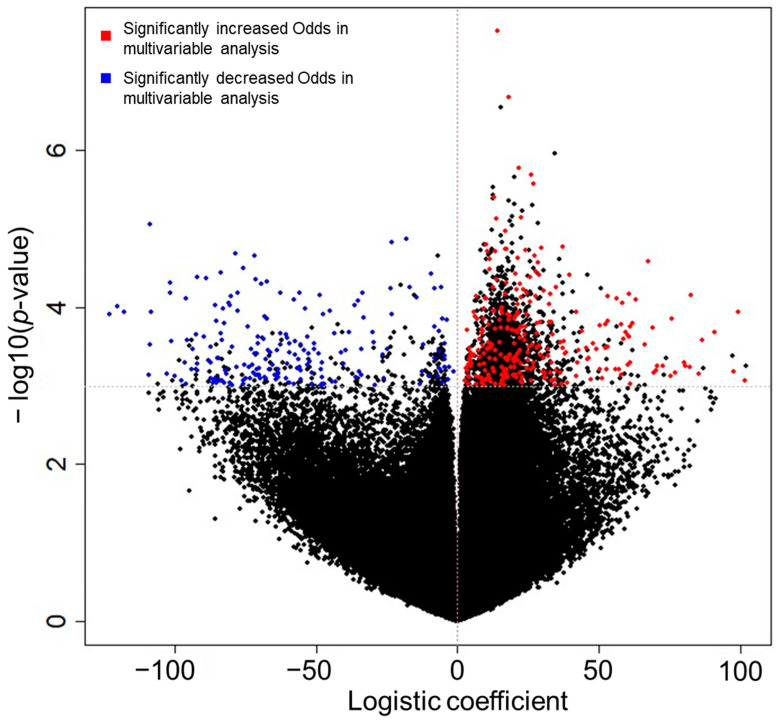
Volcano plots for the odds of CKD development found in the CKD prediction analysis. The X-axis and Y-axis represent natural log-transformed odds ratios and log-transformed *p* values, respectively. Black dots represent the results from unadjusted logistic regression and the dots plotted above than horizontal dashed line represent statistically significant values (*p* < 0.001). Blue and red dots represent significant CpGs in the multivariable-adjusted analysis, and they indicate decreased and increased odds for CKD development according to elevated methylation at each site, respectively.

During the 8-year follow-up period, CKD developed in 67 (15.2%) patients. We obtained and assessed methylation profiles, including 403,129 CpGs of 440 participants, to find the genes associated with CKD development. In the univariable logistic regression analysis, 2187 CpGs were associated with the risk of CKD development, and 457 CpGs remained significant in the multivariable logistic regression analysis adjusted with age, sex, baseline eGFR, hypertension, diabetes mellitus, and serum albumin level. Blue and red dots in the volcano plot in Figure 2 represent decreased and increased odds for CKD development with increasing methylation at each site found in the multivariable analysis. Among the 457 significant CpGs, increased methylation in 268 CpGs was associated with a greater risk of CKD, while increased methylation in the other 189 CpGs correlated with a decreased risk of CKD. Among the significant CpG sites in the CKD prediction analysis, CpGs included in specific genomes are shown in Table S1. 

### 3.3. Relationship between Methylation and eGFR Changes over Time (Kidney Function Slope Analysis)

Among the 440 participants, second follow-up methylation profiles were examined in 49 participants after 8 years. To find the association between changes in eGFR and methylation status, we calculated the percent change in eGFR as well as percent changes in methylation levels from baseline to follow-up. Among the 431,651 CpGs that remained after filtering for quality control, the change in methylation levels in 1181 CpGs was associated with the change in eGFR in the univariable linear regression analysis. In the multivariable linear regression analysis adjusted with age, sex, baseline eGFR, hypertension, diabetes mellitus, and serum albumin level, 913 CpGs still continued to have a significant relationship with eGFR changes. Increased and decreased methylation levels in 298 and 615 CpGs from baseline to 8 years of follow-up were associated with a rapid decline in eGFR over time, respectively. Among the significant CpG sites in kidney function slope analysis, CpGs included in specific genomes are shown in Table S2.

### 3.4. Functional Enrichment Features and Common Significant CpG Sites in CKD Prediction and Kidney Function Slope Analyses

**Figure 3:**
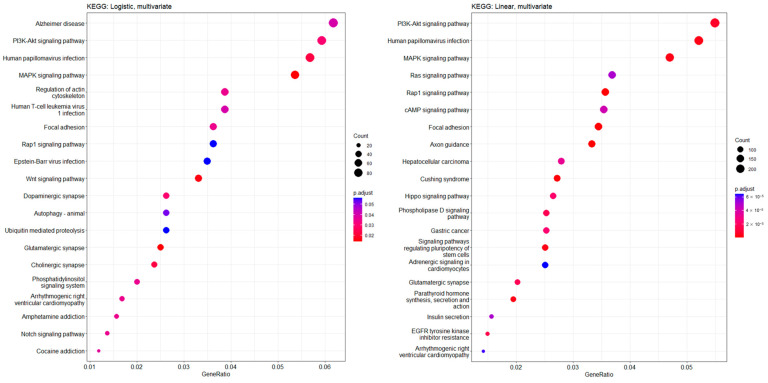
KEGG cluster profiles in CKD prediction and kidney function slope analyses. From the top, the top 20 KEGG pathways correlated with CKD prediction (**left**) and kidney function slope (**right**) results were listed. Dot size represents the gene count related to specific pathways and dot color represents adjusted *p* values. X-axis indicates the gene ratio, which was calculated by the count of the selected genes in this analysis/count of pathway genes.

We further performed a functional enrichment profile analysis with Kyoto Encyclopedia of Genes and Genomes (KEGG) pathways related to significant CpGs found in the CKD prediction and kidney function slope analyses, respectively (Figure 3). In the top 20 significant KEGG pathways, multiple pathways related to signal transduction, such as MAPK signaling, PI3K/Akt signaling, and Rap1 signaling pathways, were commonly included in both analyses.

**Figure 4:**
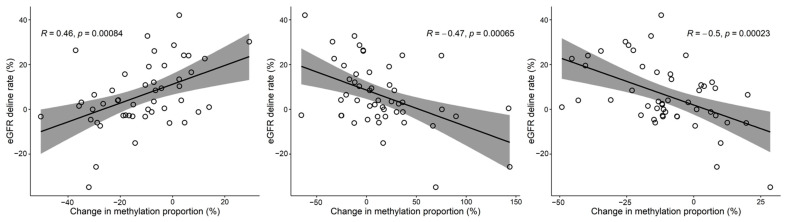
Scatter plots and linear regression lines illustrate the methylation changes at three CpG sites of three genes (NPHS2, CHCHD4, and ARH from left to right) associated with the progression of renal dysfunction and the development of CKD. The solid black lines represent the fitted linear regression lines, and the gray areas indicate the 95% confidence intervals. The x-axis represents the percentage change in methylation from baseline to follow-up measurements: (follow-up methylation—baseline methylation)/baseline methylation × 100. The y-axis represents the percentage decline in eGFR from baseline to follow-up measurements: (baseline eGFR—follow-up measurement eGFR)/baseline eGFR × 100.

We combined the results from CKD prediction and kidney function slope analyses to find common CpG sites for both analyses. Three CpGs (cg13931925, cg16463573, and cg16535332) were consistently significant in both analyses (cg13931925: multivariable logistic q = 0.043 and multivariable linear regression q = 0.019; cg16463573: multivariable logistic q = 0.017 and multivariable linear regression q = 0.04; cg16535332: multivariable logistic q = 0.027 and multivariable linear regression q = 0.026). We found three genes, NPHS2, CHCHD4 and AHR, associated with these CpG sites, which were significantly associated with the decline in kidney function. Greater methylation of CpG sites in NPHS2 at baseline was associated with a higher risk of CKD development during follow-up, and the increased methylation level of CpG sites in NPHS2 for 8 years correlated with a more rapid decline in eGFR. On the other hand, the methylation of CpGs in CHCHD4 and AHR showed an opposite relationship with NPHS2 in CKD development and the degree of eGFR decline (Figure 4). That is, hypermethylation of CHCH4 and AHR was not only associated with a lower risk of developing CKD, but was also associated with slower eGFR decline. In addition, combined results for each significant CpG site were plotted on a Circos genomic map (Figure S1). 

## 4. Discussion

In this study, using two different methods we demonstrated that DNA methylation profiles were significantly associated with CKD. Methylation sites associated with MAPK, PI3K, and Rap1 signaling pathways were commonly found in both analyses. We discovered that the degree of methylation and changes over time of three genes, *NPHS2, CHCHD4*, and *AHR* were associated with both the development and progression of CKD. 

DNA methylation, in concert with other regulators, is a major epigenetic factor influencing gene activities [30,31]. Methylation of CpG motifs in promotor regions is generally associated with transcription repression. Previous epigenome-wide association studies (EWAS) have identified compound correlations between DNA methylation levels at individual CpG sites and diseases such as cancer [32,33], type II diabetes [34], and Alzheimer’s disease [31,35].

In the area of kidney disease, EWAS have also been conducted, but their sample sizes and longitudinal study design are limited. In a Swedish study of global methylation in 155 CKD patients compared to 36 healthy controls, no association was found between the total DNA methylation ratio and eGFR [36]. In another cross-sectional study comparing 255 CKD and 152 controls using the Infinium^®^ HumanMethylation450 BeadChip system (Illumina, San Diego, CA, USA), 23 differentially methylated CpG sites were identified in blood samples of 255 CKD participants compared to controls [37]. Another study compared baseline methylation status between participants with rapid disease progression and stable kidney function but involved only a small number of participants (20 participants in each group) [16]. Furthermore, the majority of participants in that study already had eGFR values below 60 mL/min/1.73 m^2^ at baseline, suggesting that they were predominantly individuals with CKD. Therefore, direct comparison of their results with our study would not be appropriate. To overcome the weakness of previous studies, we adopted a longitudinal design study that could estimate the causal relationship between methylation and kidney function more specifically, as well as evaluated two consecutive methylation statuses in the same participants to ensure consistency and reliability of the results.

In the present study, we demonstrated significant CpG sites of three genes. The first gene, *NPHS2*, is located on chromosome 1q25-q31 and was initially mapped through linkage analysis in families affected by autosomal recessive steroid-resistant nephrotic syndrome [38]. This gene is responsible for encoding the 42 kD integral membrane protein known as podocin, which is expressed in both fetal and mature kidneys. Podocin is situated at the foot process of podocytes within the slit diaphragm, a critical site governing the filtration’s size and charge selectivity [39]. Podocin-deficient *NPHS2* knockout mice present with massive proteinuria at birth [40]. In humans, it was reported that autosomal-recessive steroid-resistant nephrotic syndrome is associated with *NPHS2* mutations, and podocin mutations result in changes in the distribution of nephrin and other proteins in podocytes [38,41]. In this study, we showed that *NPHS2* is associated with podocin dysfunction not only by mutation but also by abnormal hypermethylation.

*AHR* (aryl hydrocarbon receptor) is another gene that has been implicated in kidney disease, although its role is not as extensively studied yet. *AHR* is a ligand-activated transcription factor that plays a crucial role in xenobiotic metabolism and the regulation of various biological processes, including immune responses and cellular differentiation [42]. Studies have shown that *AHR* activation is involved in the pathogenesis of several renal diseases, including renal fibrosis and glomerular injury. 

Studies using animal models have demonstrated the involvement of *AHR* in kidney injury and disease progression. For example, in a mouse model of unilateral ureteral obstruction, *AHR* activation was shown to exacerbate renal inflammation and fibrosis, whereas *AHR* deficiency attenuated these pathological changes [43]. Furthermore, in a murine model of diabetic nephropathy, *AHR* activation contributed to macrophage infiltration, extracellular matrix accumulation and mesangial cell activation [44]. In human studies, *AHR* has been associated with kidney-related outcomes. In a cohort of patients with diabetic nephropathy, higher *AHR* expression levels were found to be associated with more severe renal pathology and worse renal function [45]. These findings suggest that *AHR* may play a role in the pathogenesis of kidney disease by contributing to inflammation, oxidative stress, and fibrosis. 

Contrary to *NPHS2* and *AHR*, which is a well-known gene involved in kidney function, the role of *CHCHD4* in kidney disease has not been studied as much. CHCHD4, a redox-sensitive mitochondrial protein, has been shown to regulate aspects of the basal cellular oxygen consumption rate, including hypoxia signaling and HIF-1α stability [46,47,48]. Stabilization of HIF-1α, a key regulator of tissue response to hypoxia in the kidneys, has been shown to ameliorate tubulointerstitial injury [49]. It was suggested to assume that methylation of *CHCHD4* might be related to a reduction in kidney function through regulating HIF-1a after hypoxic injury [50]. 

In pathway analyses, we found common associations with PI3K/Akt, MAPK, and Rap1 pathways in both analyses. Evidence for the role of the PI3K/Akt signaling pathway in renal damage has been offered by recent studies. One study reported that down-regulation of the PI3K/Akt signaling pathway reduces profibrotic interstitial cells and the potential number of tubular cells, which is associated with excessive interstitial matrix production [51]. Another study revealed that pharmacologic inhibition of PI3K reduced proliferation in fibroblasts in rat unilateral ureteral obstruction models [52]. In addition, alteration of MAPK signaling with activation by Ras or Rap1 has been reported in various kidney diseases [53,54,55]. In a unilateral ureteral obstruction model, NF-kB and MAPK signal pathways are activated to switch on the inflammatory response to aggravate kidney fibrosis [55]. One study demonstrated in vivo and in vitro ischemic–reperfusion injury induced an increase in phospho-MAPK, and fibrosis markers were decreased following the addition of MAPK inhibitor [54]. Consistent with these previous studies, the present study suggested that epigenetic changes in genes related to these pathways may be associated with renal dysfunction in the general population.

To our knowledge, this is the first study to evaluate the association between specific-site DNA methylation and CKD with serially obtained methylation datasets. Previous studies on DNA methylation have made efforts to identify biomarkers for chronic kidney disease. However, to the best of our knowledge, no study has investigated the association between methylation changes in both the initial assessment and follow-up examinations and changes in renal function, as our study did. In our research, we first identified methylation sites associated with the occurrence of CKD in a general population. Among these sites, we selected those that exhibited concurrent changes in methylation levels along with changes in renal function over time. Essentially, by employing two distinct approaches—CKD occurrence and progression—we were able to pinpoint common methylated sites. This enabled us to uncover more reliable indicators of the association between methylation and renal function.

Although our study provided novel evidence of potential epigenomic biomarkers for CKD, it is important to acknowledge several limitations. In our study, we utilized a less conservative approach in the initial screening stage, known as univariable analysis, by setting a threshold of *p* < 0.001, which is higher than the typical significance level in epigenetic studies. This decision was made to validate genes that were consistently identified across two different designs and were concomitant. However, this less conservative approach may raise concerns regarding the statistical robustness of the findings. Furthermore, it is important to note that the DNA methylation analysis conducted in our study was performed on whole blood samples rather than kidney tissue. However, emerging research suggests that differentially methylated regions relevant to specific traits in blood may exhibit similar associations in the target tissues, as supported by recent publications [56,57]. A previous study has reported associations between DNA methylation of certain CpG sites related to eGFR in blood cells and renal fibrosis when DNA methylation was measured in renal tissue [58]. This suggests a potential translation of DNA methylation patterns observed in blood to kidney tissue. This assumption finds support in the physiological role of the kidneys, which involves the filtration of waste products from the blood, as well as the involvement of immune cell-related pathophysiology in certain kidney diseases. Another limitation of our study is the potential for unadjusted variables in the multivariable adjustment analysis, which could introduce residual confounding and impact the observed associations. In addition, DNA methylation was quantified in whole blood, which represents a mixture of various cell types. It is important to acknowledge that DNA methylation patterns can vary across different cell types [59], and studying methylation in whole blood may not capture cell type-specific differences. Further investigations are warranted to address these limitations and explore the cell type-specific DNA methylation patterns associated with kidney disease in future studies. Since this study was conducted only in Korea, its application in other ethnic groups may not be suitable. Further investigations using a larger number of CpG sites and various ethnicities in larger populations are needed to allow specific epigenetic biomarkers to be established. 

## 5. Conclusions

Epigenomic and transcriptomic profiling, coupled with protein and metabolite analysis, may give a more complete picture of CKD. Genomic and epigenomic biomarkers may not only provide information on the etiology and mechanisms underlying CKD progression but may also be used for early diagnosis and appropriate treatment selection, enabling therapy to be personalized. Through this effort to move daily practice toward a precision-medicine approach, early identification of high-risk populations for CKD could ultimately guide the clinician towards a better assessment of manageable kidney risk factors and to improve patient outcomes. 

## Figures and Tables

**Table 1:** Baseline characteristics of the study participants (n = 440).

Baseline Variables	Values
Age, years [M ± SE]	52.1 ± 8.4
Female sex, [abs (%)]	215 (48.9)
Body mass index, kg/m^2^ [M ± SE]	24.6 ± 3.4
Smoking tobacco, [abs (%)]	
Current	121 (27.5)
Ex	65 (14.8)
Never	245 (55.7)
No response	9 (2.1)
Alcohol consumption, [abs (%)]	
Current	218 (49.6)
Ex	30 (6.8)
Never	186 (42.3)
No response	6 (1.4)
Hypertension, [abs (%)]	63 (14.3)
Diabetes mellitus, [abs (%)]	43 (9.8)
Dyslipidemia, [abs (%)]	7 (1.6)
Myocardial infarction, [abs (%)]	1 (0.2)
Congestive heart failure, [abs (%)]	1 (0.2)
Cerebrovascular disease, [abs (%)]	4 (0.9)
Systolic blood pressure, mmHg [M ± SE]	121.9 ± 17.8
Diastolic blood pressure, mmHg [M ± SE]	81.2 ± 11.2
eGFR CKD-EPI, mL/min/1.73 m^2^ [M ± SE]	91.8 ± 12.8
Serum blood urea nitrogen, mg/dL [M ± SE]	14.4 ± 3.4
Serum creatinine, mg/dL [M ± SE]	0.85 ± 0.17
Serum total cholesterol, mg/dL [M ± SE]	193.3 ± 35.0
Blood hemoglobin, g/dL [M ± SE]	13.7 ± 1.6
Serum albumin, g/dL [M ± SE]	4.3 ± 0.4

Abbreviation: eGFR CKD-EPI, estimated glomerular filtration rate calculated with the Chronic Kidney Disease Epidemiology Collaboration equation.

## Data Availability

The datasets used and/or analyzed during the current study are available from the corresponding author on reasonable request.

## Supplementary Materials

The following supporting information can be downloaded at: https://www.mdpi.com/article/10.3390/genes14071489/s1, Figure S1: Genomic information mapping of CpG sites for 457 in logistic regression results and 913 in linear regression results was presented as circos plot; Table S1: Significant CpGs in CKD prediction analysis; Table S2: Significant CpGs in kidney function slope analysis.
