# Supplementary material for: The Effect of DNA Methylation in the Development and Progression of Chronic Kidney Disease in the General Population: An Epigenome-Wide Association Study Using the Korean Genome and Epidemiology Study Database
Source: Genes (Basel). 2023 Jul 21;14(7):1489. doi: 10.3390/genes14071489 (PMC10379047; doi:10.3390/genes14071489)

Figure S1. Genomic information mapping of CpG sites for 457 in logistic regression results and 913 in linear regression results was presented as Circos plot. Univariable and multivariable linear regression results were plotted on 1st and 2nd tracks from outside, respectively. And univariable and multivariable logistic regression results were plotted on 3rd and 4th tracks from outside, respectively. Heatmaps in Circos plot represent regression coefficients and peaks represent p-values transformed with -log10. The labels 1-22, X, and Y on the outermost ring correspond to each chromosome.

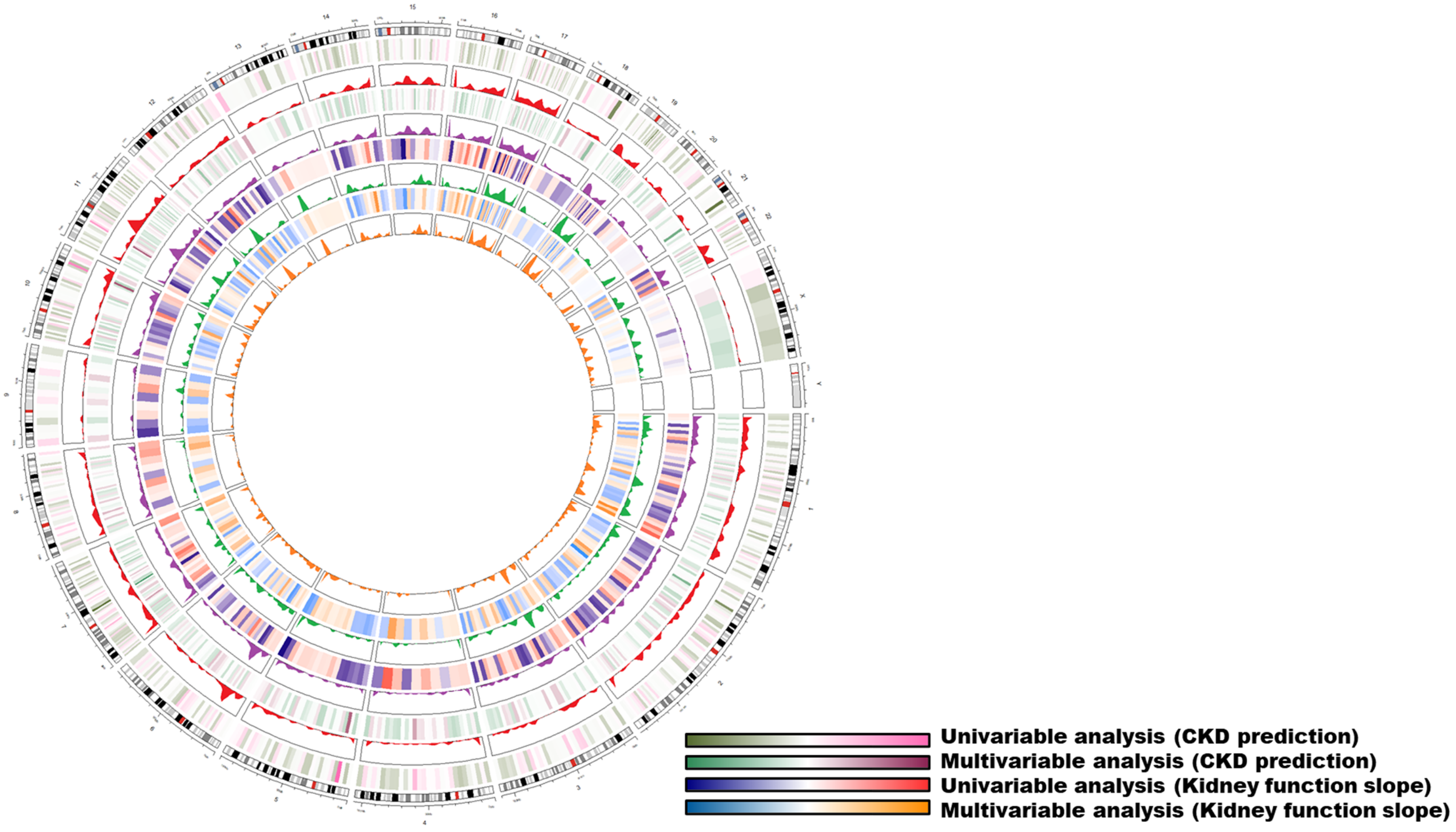

Supplement: Supplementary file 1 [file genes-14-01489-s001.zip › genes-2477815-supplementary.pdf]
